# Oxadiazole-Based Highly Efficient Bipolar Fluorescent Emitters for Organic Light-Emitting Diodes

**DOI:** 10.3390/molecules23040843

**Published:** 2018-04-07

**Authors:** Qiong Wu, Ramanaskanda Braveenth, Heng Qiang Zhang, Il-Ji Bae, Miyoung Kim, Kyu Yun Chai

**Affiliations:** 1 Division of Bio-Nanochemistry, College of Natural Sciences, Wonkwang University Iksan City, Chonbuk 570-749, Korea; wuqiong9308@126.com (Q.W.); braveenth.czbt@gmail.com (R.B.); 2 Department of Chemistry, Hebei Normal University for Nationalities, Chengde 067000, China; zhanghengqiang80@163.com; 3 Nano-Convergence Research Center, Korea Electronics Technology Institute, Jeonju 54853, Korea; ijbae@keti.re.kr

**Keywords:** bipolar, oxadiazole, acridine, phenothiazine, organic light-emitting diodes, fluorescent

## Abstract

In this study, a series of bipolar fluorescence emitters named 2DPAc-OXD, DPAc-OXD, 2PTZ-OXD and PTZ-OXD were designed and synthesized with excellent yields. The characterization of materials was investigated by using nuclear magnetic resonance (NMR) (^1^H, ^13^C), mass spectrometry and thermogravimetric analysis (TGA). To investigate device efficiencies, two different OLED devices (Device 1, Device 2) were fabricated with two different host materials (Bepp_2_, DPEPO). The Device 2 with 2PTZ-OXD as fluorescent emitter exhibited excellent power and current efficiencies of 6.88 Lm/W and 10.10 cd/A, respectively. The external quantum efficiency of 2PTZ-OXD was around 3.99% for Device 2. The overall device properties of phenothiazine donor were better than acridine derivatives.

## 1. Introduction

Organic light-emitting diodes (OLEDs) have developed rapidly since the first efficient double layer structure device was reported by Tang [1]. They are expected as the promising candidates for next generation flat-panel displays and solid-state lighting sources due to various advantages, such as high-quality color, low energy cost, light weight, and flexibility [2]. Small OLED displays, which are commercially available now, are applied to mobile displays, television and flat panels. Nevertheless, there are still some issues related to device performance and reliability need to improve, especially the efficiency of device with fluorescent emitting materials. To the best of our knowledge, the maximum external quantum efficiency (EQE) of fluorescent material is 8.5% [3]. The emitting layer functions as the recombination site of holes and electrons which are injected from anode and cathode, respectively [4]. Therefore, the development of high efficient fluorescent-emitting material is a key issue for high performance OLED devices.

The charge-recombination factor is dominated by the balance of holes and electrons in the emissive layer and the current balance is directly proportional to the emission [5]. Therefore, emitting layer materials should meet the requirements of energy level matching for charge carrier injection and acceptance of both holes and electrons. Meanwhile, they should possess the properties of electron transporting and hole-transporting character to permit the formation of both stable cation and anion radicals, which known as bipolar in nature [4]. Electron-donating units have the potential to transport holes, while electron-accepting units are capable of transporting electrons. Therefore, the most prevalent strategy of achieving bipolar materials is the incorporation of donor-acceptor (D-A) units into the same molecule via a linker to facilitate the injection and transport of both holes and electrons [6]. Recently, A-D and D-A-D types materials are often explored for OLED fluorescent materials [3,7,8,9,10,11,12,13,14,15,16] to improve charge balance and reduce driving voltage; they are also widely designed for thermally activated delayed fluorescence (TADF) emitters and host martials for phosphorescence and TADF dopants [17,18,19]. The emission from A-D and D-A-D type molecular design can originate from intramolecular charge-transfer (ICT) excited states between the donor and acceptor moieties [20,21]. Therefore, judicious combination of the appropriate donor and accepter could facilitate simultaneous manipulation of the highest occupied orbital (HOMO)/the lowest unoccupied orbital (LUMO) levels and the emission color of the bipolar structure molecule [22].

Triazine, oxadiazole, triazole, cyno-substituted benzene and benzothiadiazole are usually used as acceptor units, while carbazole, arylamine, phenothiazine and their derivatives are commonly adopted as the donor moieties for bipolar structures for OLEDs [15,16,17,20,23,24,25,26,27,28,29]. Herein, we utilize phenothiazine and 9,9-diphenyl-9,10-dihydroacridine as the donor unit, while oxadiazole derivatives 2,5-bis(4-bromophenyl)-1,3,4-oxadiazole and 2-(4-bromophenyl)-5-phenyl-1,3,4-oxadiazole are employed as the acceptors to synthesize four distorted A-D and D-A-D type fluorescent materials, 2,5-bis(4-(10H-phenothiazin-10-yl)-1,3,4-oxadiazole (2PTZ-OXD), 2-(4-(10H-phenothiazin-10-yl)-1,3,4-oxadizaole (PTZ-OXD), 2,5-bis(4-(9,9-diphenyl-9,10-dihydroacridine)phenyl)-1,3,4-oxadizaole (2DPAc-OXD) and 2-(4-(9,9-diphenyl-9,10-dihydroacridine)phenyl)-1,3,4-oxadiazole (DPAc-OXD). We also investigated their photophysical properties, thermal stability and performed quantum calculations to evaluate the molecular orbitals distribution for all molecules.

## 2. Results and Discussion

### 2.1. Synthesis

**Scheme 1:**
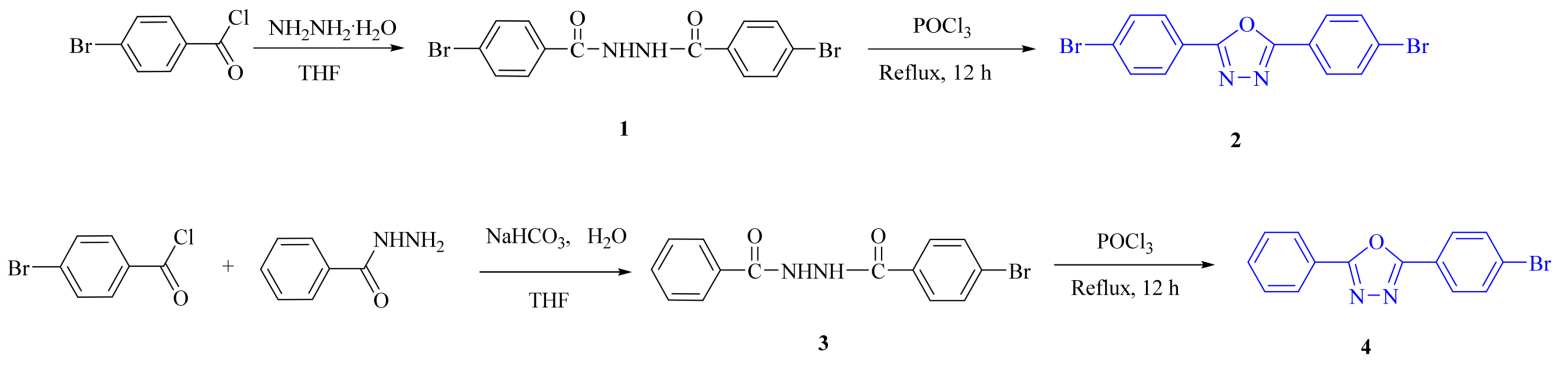

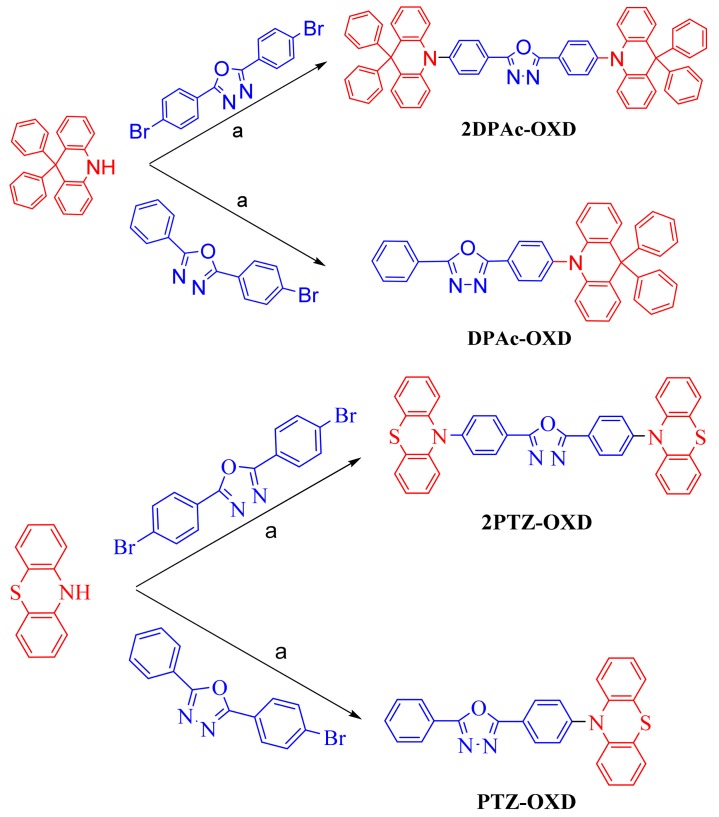
Synthesis of 2,5-bis(4-(9,9-diphenyl-9,10-dihydroacridine)phenyl)-1,3,4-oxadizaole (2DPAc-OXD), 2-(4-(9,9-diphenyl-9,10-dihydroacridine)phenyl)-1,3,4-oxadiazole (DPAc-OXD), 2,5-bis(4-(10H-phenothiazin-10-yl)-1,3,4-oxadiazole (2PTZ-OXD) and 2-(4-(10*H*-phenothiazin-10-yl)-1,3,4-oxadizaole (PTZ-OXD). Reagents and conditions (a): Pd(OAc)_2_, K_2_CO_3_, *t*-Bu_3_P, anhydrous toluene, 110 °C.

Scheme 1 displays the synthetic process of designed bipolar fluorescent emitters. Compounds **1**, **2**, **3** and **4** were known and synthesized by following the methods from previously reported literatures [30,31,32]. 2DPAc-OXD, DPAc-OXD, 2PTZ-OXD and PTZ-OXD were synthesized by using the well-known Buchwald Hartwig cross-coupling reaction between the oxadiazole (2 and 4) and phenothiazine or 9,9-diphenyl-9,10-dihydroacridine derivatives by using palladium-based catalyst.

### 2.2. Thermal Properties

**Figure 1:**
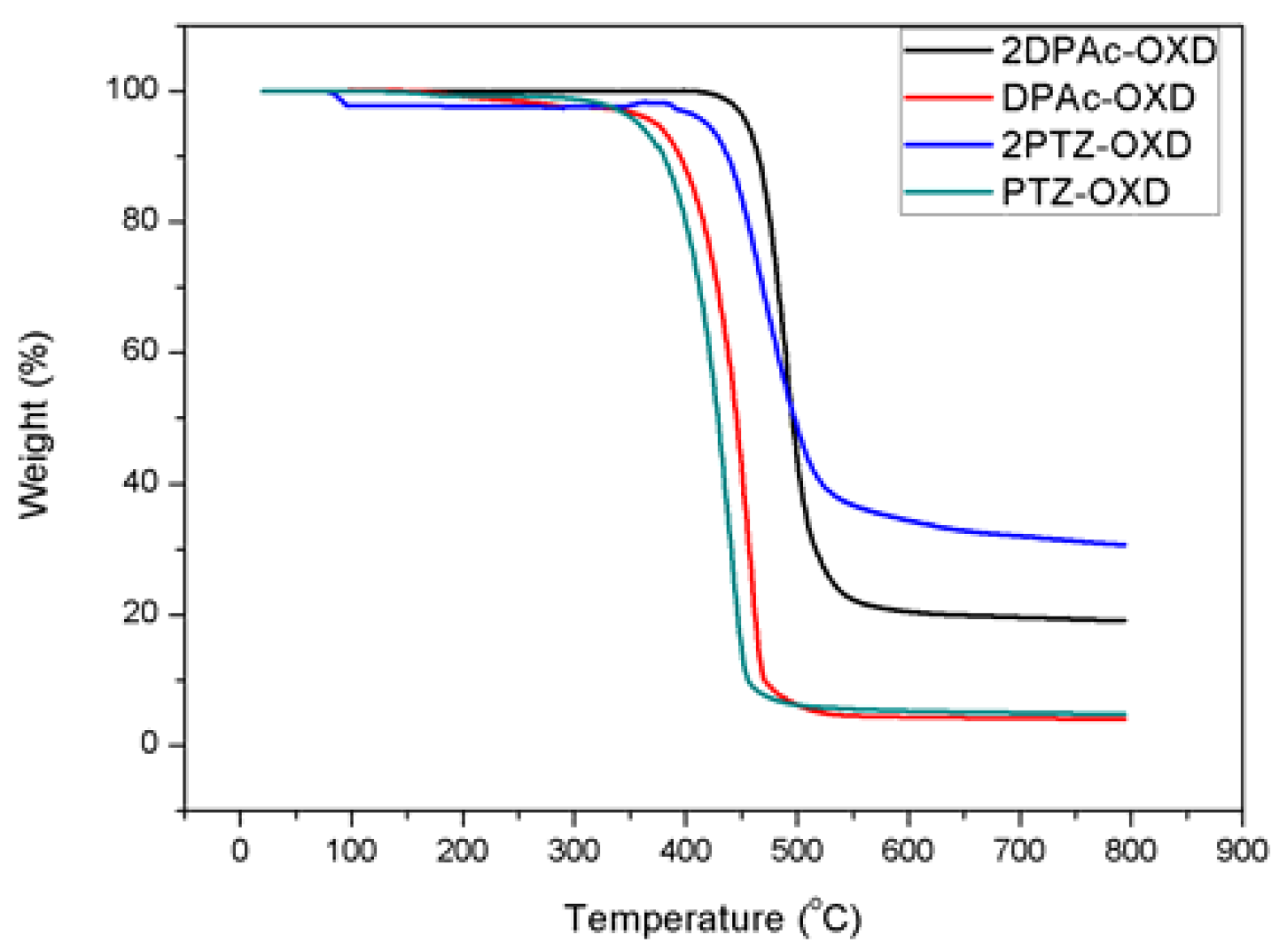
Thermal decomposition temperature (by thermogravimetric analysis, TGA) of fluorescent emitters.

The thermal stabilities of four fluorescence emitters were studied on the basis thermal decomposition temperature at 5% weight reduction and were measured by using thermo gravimetric analysis (TGA). All four emitters, which are depicted in Figure 1 exhibited higher decomposition temperature over 358 °C. The acridine donor-based emitters, DPAc-OXD and 2DPAc-OXD showed thermal stability of 375 and 455 °C, respectively. The above values are higher than that of phenothiazine donor based PTZ-OXD (358 °C) and 2PTZ-OXD (419 °C) due to its higher rigidity when compare to acridine donor-based molecules (Table 1). Additionally, we noticed that two site molecules (2PTZ-OXD, 2DPAc-OXD) were excellent with their thermal strength while the thermal strength of single site molecules (PTZ-OXD, DPAc-OXD) were lower.

### 2.3. Photophysical and Electrochemical Properties

**Figure 2:**
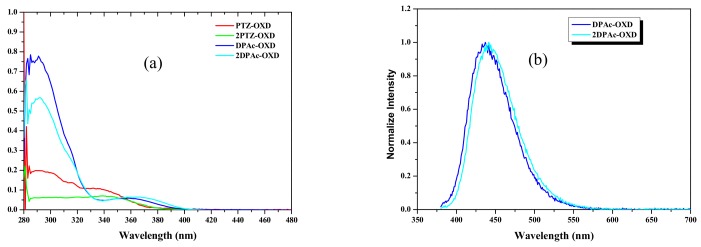
(**a**) UV-Vis absorption spectra (**b**) Photoluminescent spectra of fluorescent emitters.

The photophysical properties were analysed by using UV-visible and photoluminescence spectral measurements. UV-Vis spectra showed on set values of 376, 384, 396 and 402 nm for PTZ-OXD, 2PTZ-OXD, DPAc-OXD and 2DPAc-OXD, respectively which were used to calculate the band gap energies of four emitters (Figure 2). The above values are summarized in Table 1. The triplet energy of PTZ-OXD, 2PTZ-OXD, DPAc-OXD and 2DPAc-OXD were 2.48, 2.42, 2.85 and 2.81 eV, respectively. Those above values were evaluated from phosphorescence emission spectrum according to literature [33]. The triplet energy values were matched with their photoluminescence (PL) emission of 500 (PTZ-OXD), 512 (2PTZ-OXD), 435 (DPAc-OXD) and 442 nm (2DPAc-OXD). When we noticed the PL values of phenothiazine donor-based molecules, PTZ-OXD and 2PTZ-OXD were revealed bathochromic shift related to their lower triplet energy. HOST material is one of the key factors for enhancing the device efficiencies by controlling holes and electrons balance at the emission layer. We have fabricated two different OLED devices with different host materials of Bis[2-(2-pyridinyl)phenolato]beryllium(II) (Bepp_2_) and Bis[2-(diphenylphosphino)phenyl] ether oxide (DPEPO), which have higher triplet energy of 2.70 and 2.99 eV for latest [34,35]. DPEPO host material has enough potential to prevent the energy transferring from dopant to host due to its higher triplet energy than our fluorescence emitters. In the case of Bepp_2_ host material, it can effectively work with PTZ-OXD and 2PTZ-OXD due to their lower triplet energies.

The electrochemical properties were analysed on the basis of HOMO and LUMO energies. The HOMO value of acridine donor based DPAC-OXD and 2DPAC-OXD were the same with −5.68 eV, while phenothiazine donor based PTZ-OXD and 2PTZ-OXD were almost identical to each other (−5.48 eV). Consequently, LUMO energy values were calculated by subtracting the band gap energies from HOMO energies and the values were −2.18, −2.25, −2.56 and −2.60 eV for PTZ-OXD, 2PTZ-OXD, DPAc-OXD and 2DPAc-OXD, respectively. The frontier molecular energies contributed to constructing the device structure substantiated with proper energy flow with adjacent layers.

**Figure 3:**
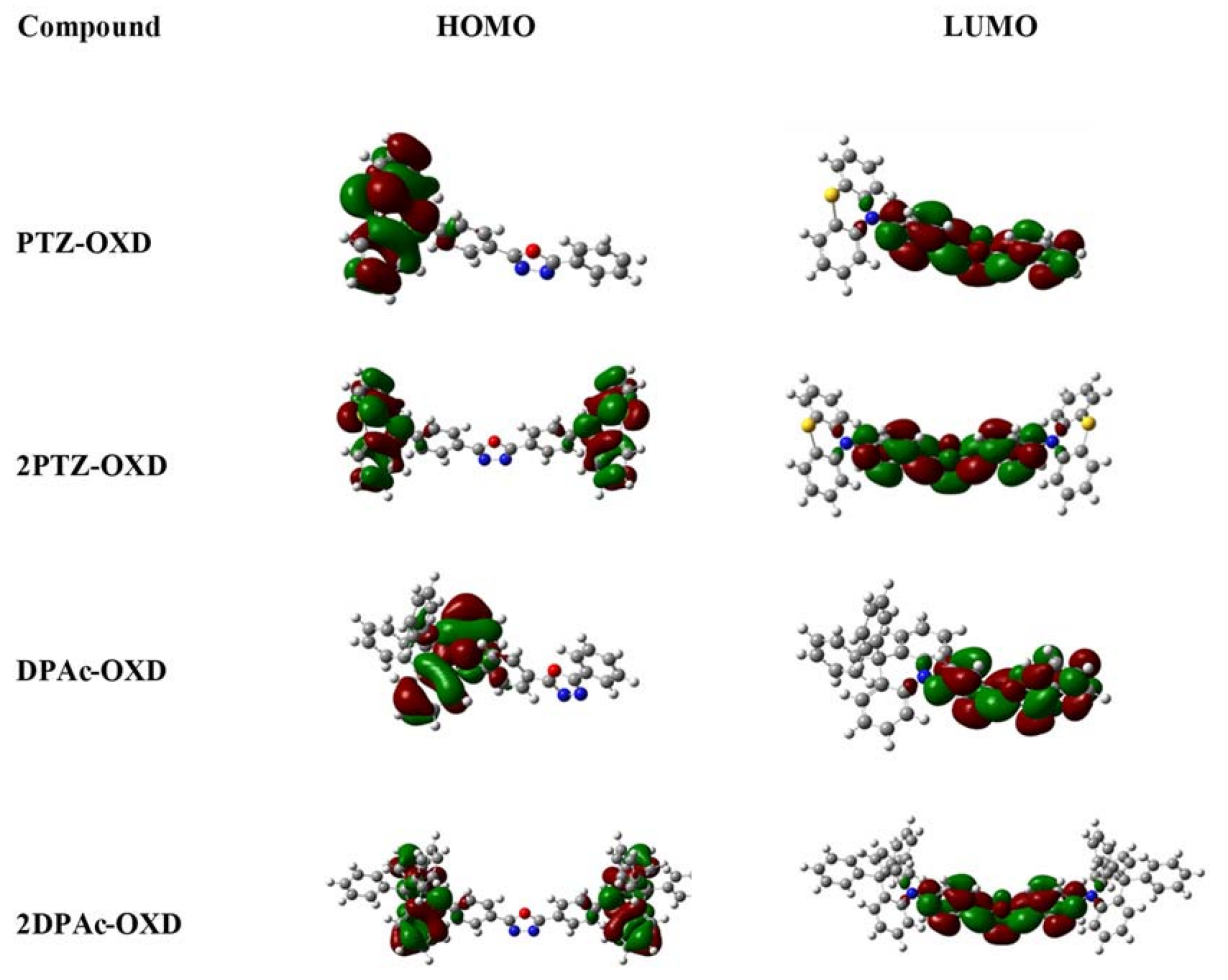
Calculated Frontier molecular orbital distribution.

Density functional theory (DFT) calculations were investigated by using Gaussian 9 program with TD-SCF method B3LYP and 6-31G basis set [36]. The frontier molecular orbital (FMO) distributions are shown in Figure 3. All four materials had clear HOMO and LUMO separations and we did not notice any overlapping between the donor and acceptor. HOMO of all molecules was distributed over phenothiazine and acridine moieties due to their strong electron donating nature, which could be explained by the electron localization on donor molecules. The electron delocalization was observed at oxadiazole due to its electron withdrawing nature. The clear separation of FMOs are supported by their lower singlet-triplet energy gap difference of 0.35, 0.29, 0.46 and 0.44 eV for PTZ-OXD, 2PTZ-OXD, DPAc-OXD and 2DPAc-OXD, respectively (Table 2). The rotation angle between phenothiazine donor and oxadiazole was around 78°, but acridine and oxadiazole showed higher angle of 81° as bulky phenyl groups attached with *sp*^3^ carbon atom of acridine molecule. The above results indicate that four A-D and D-A-D type fluorescence emitters were successfully constructed with bipolar charge transfer properties.

### 2.4. Device Characteristics

**Figure 4:**
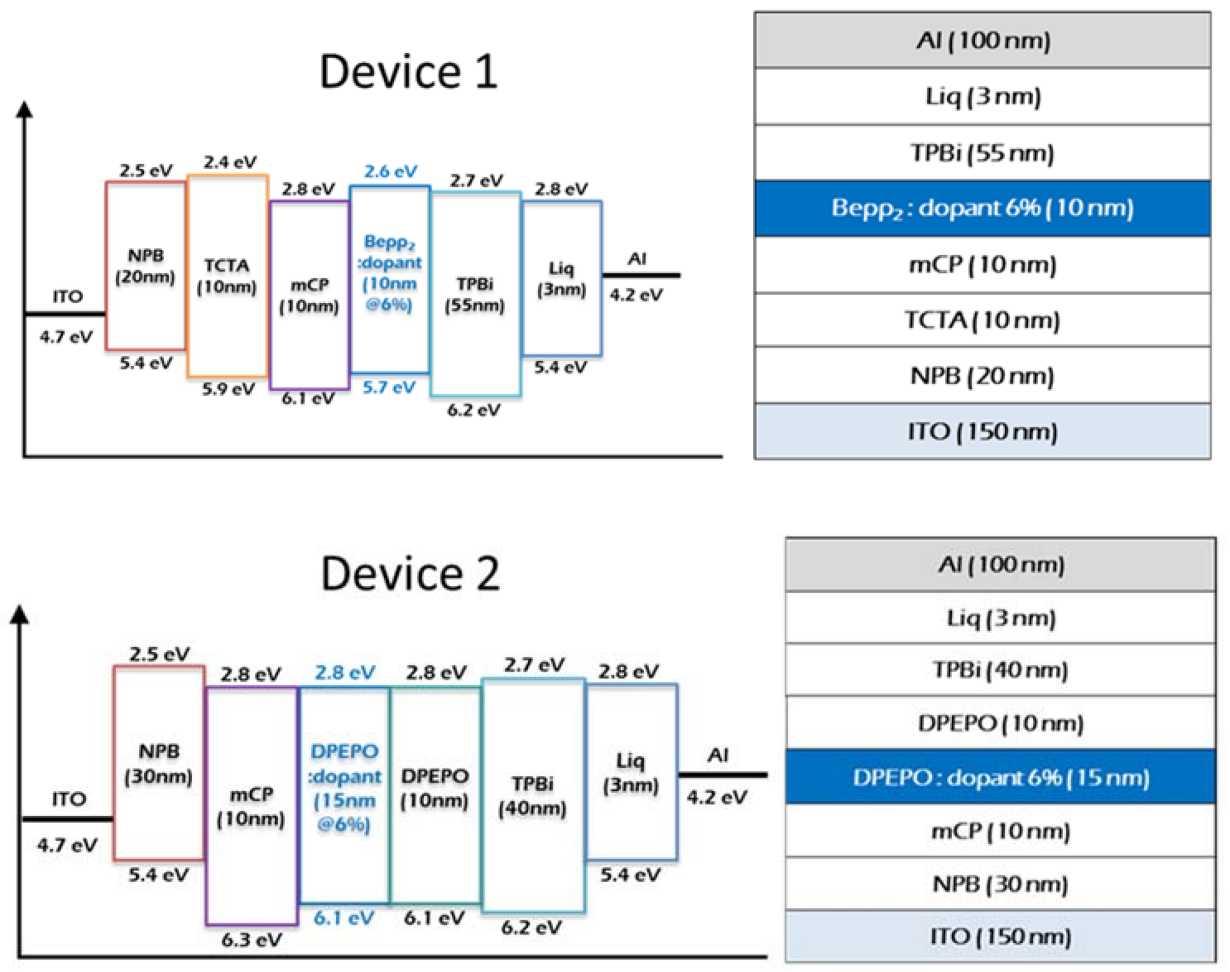
The fabricated device structures of 1 and 2 based on fluorescent emitters.

The device characteristics were studied after fabricating OLED devices with two different host materials (Figure 4). The device structures were (Device 1) ITO (150nm)/ NPB (20 nm)/ TCTA (10 nm)/ mCP (10 nm)/ Bepp_2_: dopant 6%(10 nm)/TPBi (55 nm)/ Liq (3 nm)/ Al (100 nm) and (Device 2) ITO (150 nm)/ NPB (30 nm)/ mCP (10 nm)/ DPEPO: dopant 6%(15 nm)/DPEPO (10 nm)/ TPBi (40 nm)/ Liq (3 nm)/ Al (100 nm).

**Figure 5:**
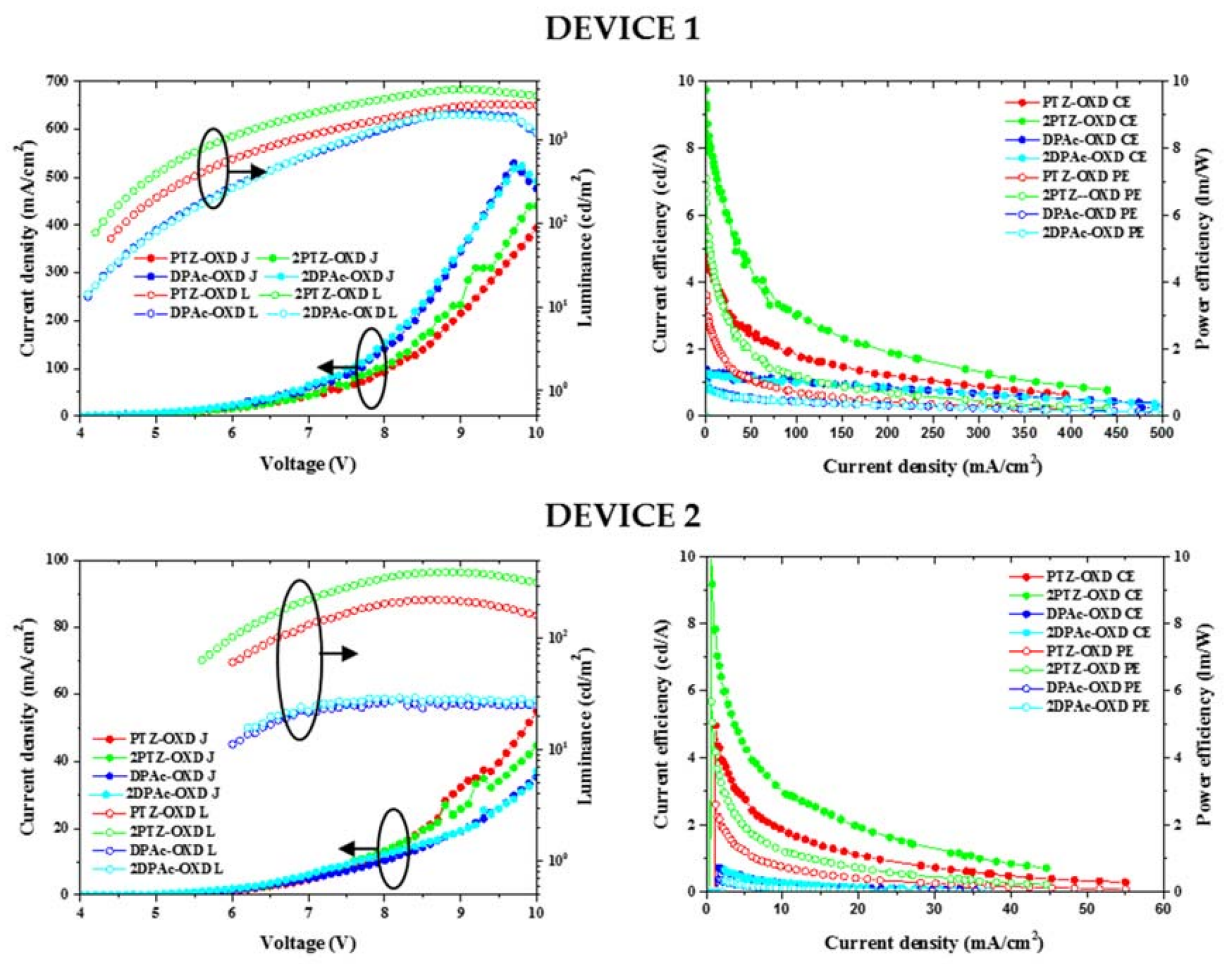
Current density-voltage-luminance (*J-V-L*) and luminescence -current and power efficiency of the fabricated Devices 1 and 2.

The turn on voltages of device 1 were between 4.1 to 4.4 V, which was lower than that of device 2 (5.6 to 6.2 V). PTZ-OXD and 2PTZ-OXD showed lower driving voltage than acridine based DPAc-OXD and 2DPAc-OXD, which proved that phenothiazine donor in cooperating with oxadiazole acceptor can enhance the device efficiencies (Figure 5). The current efficiency of device 1 was better than device 2, but 2PTZ-OXD based Device 1 exhibited current efficiency of 9.20 cd/A while Device 2 was 10.10 cd/A. The above value is higher than reported current efficiency of fluorescence-based emitters in OLEDs. 2PTZ-OXD based Device 1 showed higher power efficiency of 6.88 Lm/W, which is related with lower driving voltage of 5.9 V. PTZ-OXD revealed power efficiency of 3.59 Lm/W for its higher driving voltage (6.8 V). The current and power efficiencies of acridine based DPAc-OXD and 2DPAc-OXD were lower than phenothiazine-based molecules which is an evidence that acridine and oxadiazole do not provide balance charge transfer system due to strong electron donating ability of acridine molecule than that of phenothiazine. Consequently, external quantum efficiency (EQE) of 2PTZ-OXD based Device 1 was 3.38% and Device 2 was 3.99%. The EQE value of PTZ-OXD, DPAc-OXD and 2DPAc-OXD were 1.94%, 1.84% and 1.81% respectively (Table 3). However, the efficiencies at high current density were dropped from peak value due to efficiency roll-off. Which attributed to quenching mechanism such as molecular exciton annihilation occurs at high current densities [37,38].

**Figure 6:**
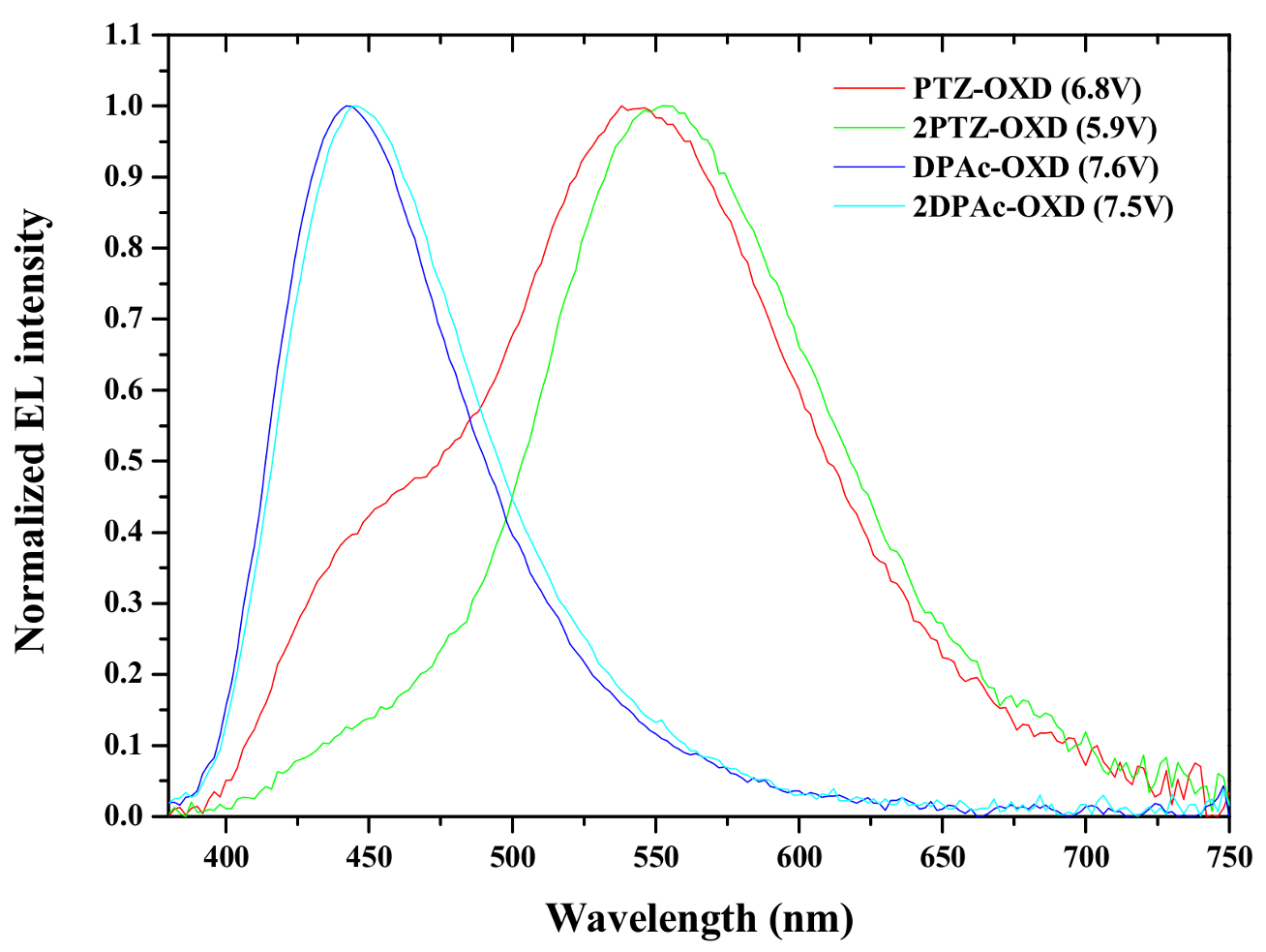
Normalized electroluminescent (EL) spectra of Device 1.

The electroluminescence study was carried out to find the emission wavelength and colour purity (Table 3). DPAc-OXD and 2DPAc-OXD showed similar emission peak at 450 nm along with CIE (x, y) colour coordinate of (0.16, 0.12) and (0.17, 0.14), respectively. The emission of PTZ-OXD was observed at 540 nm, while 2PTZ-OXD showed emission at 550 nm with little bathochromic shift. We did not notice any other emissions other than our fluorescence dopants (Figure 6).

## 3. Materials and Methods

### 3.1. General Procedures

All reagents and solvent were purchased from commercial suppliers and were used without further purification otherwise stated. ^1^H- and ^13^C-NMR (Nuclear Magnetic resonance) spectra were recorded by using a JNM-ECP FT-NMR spectrometer (JEOL, Peabody, MA, USA) and operating at 500 MHz. Absorbance spectra were recorded from a S-4100 UV-visible spectrophotometer (SINCO, Seoul, Korea). The Band gaps (*E*_g_) were estimated from the onset of the absorbance spectra while photoluminescence (PL) spectra were measured by using a HR800 Spectro fluorimeter (Horiba Jobin Yvon, Paris, France). HOMO level was calculated by AC-2 using a Photoelectron spectrometer (RIKEN, Saitama, Japan). LUMO was calculated by subtraction of the band gap from the HOMO energy. Thermal gravimetric analysis was conducted on a DSC Q200 V24.9 Build 121 thermal analysis system (TA instruments, New castle, DE, USA) with the heating rate of 10 ^°^C/min. Mass analysis were carried out by using a Xevo TQ-S spectrometer (Waters, Milford, MA, USA). Current density-voltage-luminescence (*J-V-L*) efficiencies were measured by an OLED I-V-L test system (Polarmix M6100, Suwon, Republic of Korea). The electroluminescence (EL) spectra analysis was carried out by using a spectroradiometer (Konica Minolta CS-2000, Japan). The molecular distributions were carried out by using a Gaussian 09 program (Wallingford, CT, USA) with DFT (density functional theory) and TD-SCF method B3LYP with a 6/31G basic set.

### 3.2. Synthetic Procedures

#### 3.2.1. 4-Bromo-*N*′-(4-bromobenzoyl)benzohydrazide (**1**)

4-Bromobenzoyl chloride (3.0 g, 13.67 mmol) was dissolved in 30 mL THF and stirred at 0 ^°^C for 30 min, then hydrazine monohydrate (0.28 mL, 5.44 mmol) was added dropwise in to the mixture. After adding, the mixture was stirred for another 3 h at room temperature. The resulting precipitate was filtered, washed with saturated aqueous NaHCO_3_ solution (3 × 15 mL) and water, respectively, and then dried to get white solid (1.94 g) with the yield of 84%.

^1^H NMR (500 MHz, DMSO-*d*_6_) δ 10.65 (s, 2H), 7.88–7.83 (m, 4H), 7.75 (dd, *J* = 6.42, 4.53 Hz, 4H).

#### 3.2.2. 2,5-Bis(4-bromophenyl)-1,3,4-oxadiazole (**2**)

4-Bromo-*N*′-(4-bromobenzoyl)benzohydrazide(5 g, 12.56 mmol) was placed in 250 mL two neck flask equipped with condenser. Then vacuumed it for 15 min and 30 mL of dry toluene and 30 mL POCl_3_ were injected. Under N_2_ atmosphere, the mixture was refluxed until the reaction was finished. After completion of the reaction, the solvent and excesses POCl_3_ were removed by a rotary evaporator under reduced pressure. Finally, the mixture was recrystallized with ethanol. The white crystals were filtered and dried (4.75 g, 99.5% yield).

^1^H NMR (500 MHz, CDCl_3_) δ 8.00 (td, *J* = 8.00, 1.72 Hz, 4H), 7.68 (td, *J* = 7.99, 1.70 Hz, 4H); ^13^C NMR (500 MHz, CDCl_3_) δ 164.15,132.59, 128.44, 126.73, 122.72.

#### 3.2.3. 2-(4-Bromophenyl)-5-phenyl-1,3,4-oxadiazole (**4**)

The synthesis of 2-(4-bromophenyl)-1,3,4-oxadiazole is similar with OXD-2Br, and the only difference lies in the starting material 4-bromo-*N*′-(4-bromobenzoyl)benzohydrazide to *N*′-benzoyl-4-bromobenzohydrazide. After recrystallization, white crystals were filtered and dried in the oven.

Yield 93%. ^1^H NMR (500 MHz, CDCl_3_) δ 8.15 (dd, *J* = 5.30, 3.40 Hz, 2H), 8.04–7.99 (m, 2H), 7.63–7.58 (m, 2H), 7.57–7.52 (m, 1H), 7.52–7.46 (m, 2H); ^13^C NMR (500 MHz, CDCl_3_) δ 144.85, 143.55, 133.66, 132.69, 132.08, 131.93, 130.09, 128.75, 128.71, 126.87.

#### 3.2.4. 2,5-Bis(4-(9,9-diphenyl-9,10-dihydroacridine)phenyl)-1,3,4-oxadiazole (**2DPAc-OXD**)

2,5-Bis(4-bromophenyl)-1,3,4-oxadiazole (5 g, 13.16 mmol), 9,9-diphenyl-9,10-dihydroacridine (9.64 g, 28.94 mmol), potassium carbonate (12.04 g, 87.09 mmol) and palladium acetate (0.18 g, 0.79 mmol) were added in 500 mL two neck flask equipped with condenser and vacuumed it for 15 min. Then 300 mL of dry toluene was injected. The mixture was continued with stirring and heating, after 10 min 7.3 mL tri-tert-butylphosphine (10% in toluene) was injected. The above mixture was stirred and heated under reflux condition until the starting material disappear. After cooling to room temperature, the mixture was extracted with dichloromethane (DCM) and deionized water. Then the organic layer was dried over anhydrous MgSO_4_ and filtered. The organic layer was concentrated on a rotary evaporator under reduced pressure. Finally, the residue was purified by silica column chromatography to afford 8.76 g of (white solid) 2DPAc-OXD with the yield of 75.2%.

^1^H NMR (500 MHz, CDCl_3_) δ 8.33–8.29 (m, 4H), 6.46 (d, *J* = 8.24 Hz, 4H), 6.92 (d, *J* = 4.01 Hz, 8H), 6.99 (dd, *J* = 7.67, 1.77 Hz, 8H), 7.08 (td, *J* = 8.38, 4.34, 4.34 Hz, 4H), 7.29–7.21 (m, 16H); ^13^C NMR (500 MHz, CDCl_3_) δ 164.36, 146.28, 144.46, 143.93, 141.83, 132.13, 130.45, 130.27, 129.27, 127.77, 127.05, 126.47, 120.78, 114.24, 108.01; MS (APCI): 886.09 for C_64_H_44_N_4_O [M+H^+^].

#### 3.2.5. 2-(4-(9,9-Diphenyl-9,10-dihydroacridine)phenyl)-1,3,4-oxadiazole (**DPAc-OXD**)

2-(4-bromophenyl)-5-phenyl-1,3,4-oxadiazole (3 g, 9.96 mmol), 9,9-diphenyl-9,10-dihydroacridine (3.65 g, 1.96 mmol), potassium carbonate (4.54 g, 32.87 mmol) and palladium acetate (0.07 g, 0.299 mmol) were added in 250 mL two neck flask equipped with condenser and subjected it for 15 min. Then 80 mL of dry toluene was injected, followed by 2.8 mL of tri-tert-butylphosphine (10% in toluene) was added. After the completion of reaction, mixture was extracted with DCM and water. The organic layer was dried over anhydrous MgSO_4_ then filtered and evaporated under reduced pressure. The residue was purified by silica column chromatography to afford 4.7 g of DPAc-OXD as white solid with the yield of 85.4%.

^1^H NMR (500 MHz, CDCl_3_) δ 8.33–8.29 (m, 2H), 8.16 (dd, *J* = 7.83, 1.71 Hz, 2H), 7.61–7.55 (m, 3H), 7.31–7.24 (m, 8H), 7.08 (ddd, *J* = 8.46, 5.57, 3.22 Hz, 2H), 7.00 (dd, *J* = 7.89, 1.70 Hz, 4H), 6.97–6.89 (m, 4H), 6.46 (d, *J* = 8.05 Hz, 2H); ^13^C NMR (500 MHz, CDCl_3_) δ 164.94, 164.13, 146.32, 144.29, 141.86, 132.12, 131.99, 130.46, 130.27, 130.20, 129.25, 127.77, 127.09, 127.05, 126.47, 123.89, 123.70, 120.74, 114.22; MS (APCI): 553.75 for C_39_H_27_N_3_O [M+H^+^].

#### 3.2.6. 2,5-Bis(4-(10*H*-phenothiazin-10-yl)-1,3,4-oxadizole (**2PTZ-OXD**)

2,5-Bis(4-bromophenyl)-1,3,4-oxadiazole (5 g, 13.15 mmol), phenothiazine (5.76 g, 28.93 mmol), potassium carbonate (12.03 g, 87.05 mmol) and palladium acetate (0.18 g, 0.79 mmol) were added in 250 mL two neck flask equipped with condenser and vacuumed it for 15 min. Then 120 mL of dry toluene was injected while stirring. Then 7.3 mL of tri-tert-butylphosphine (10% in toluene) was injected in to the mixture. The above mixture was stirred and refluxed. After reaction completed, mixture was cooled to room temperature and extracted with dichloromethane (DCM) and deionized water. Then the organic layer was dried over MgSO_4_, followed by concentrated on a rotary evaporator under reduced pressure. Finally, the residue was purified by silica column chromatography to afford 7.88 g (yellow solid) 2PTZ-OXD with the yield of 97.2%.

^1^H NMR (500 MHz, CDCl_3_) δ 8.17–8.13 (m, 2H), 7.38–7.33 (m, 2H), 7.28–7.22 (m, 2H), 7.12 (dt, *J* = 7.75, 7.66, 1.50 Hz, 2H), 7.05–7.02 (m, 2H), 6.84 (d, *J* = 8.07 Hz, 2H); ^13^C NMR (500 MHz, CDCl_3_) δ 160.15, 146.50, 142.60, 128.88, 128.03, 127.47, 127.23, 124.65, 123.80, 121.54, 120.08; MS (APCI): 618.07 for C_38_H_24_N_4_OS_2_ [M+H^+^].

#### 3.2.7. 2-(4-(10*H*-phenothiazin-10-yl)-1,3,4-oxadiazole(**PTZ-OXD**)

2-(4-Bromophenyl)-5-phenyl-1,3,4-oxadiazole (3 g, 9.96 mmol), phenothiazine (2.18 g, 10.96 mmol), potassium carbonate (4.56 g, 32.98 mmol) and palladium acetate (0.07 g, 0.299 mmol) were added in 250 mL two neck flask equipped with condenser and vacuumed it for 15 min. Then 80 mL dry toluene was injected while stirring and 2.8 mL tri-tert-butylphosphine (10% in toluene) was added. The mixture was stirred and refluxed until the reaction complete. After cooling to room temperature, the mixture was extracted with dichloromethane (DCM) and deionized water. The organic layer was dried over MgSO_4_, followed by concentrated on a rotary evaporator under reduced pressure. Finally, the residue was purified by silica column chromatography afford 4.18 g (light yellow solid) of PTZ-OXD with the yield of 98.5%.

^1^H NMR (500 MHz, CDCl_3_) δ 8.33–8.29 (m, 2H), 8.16 (dd, *J* = 7.83, 1.71 Hz, 2H), 7.61–7.55 (m, 3H), 7.31–7.24 (m, 8H), 7.08 (ddd, *J* = 8.46, 5.57, 3.22 Hz, 2H), 7.00 (dd, *J* = 7.89, 1.70 Hz, 4H), 6.97–6.89 (m, 4H), 6.46 (d, *J* = 8.05 Hz, 2H); ^13^C NMR (500 MHz, CDCl_3_) δ 164.94, 164.13, 146.32, 141.86, 132.12, 131.99, 130.47, 130.27, 129.25, 127.78, 127.09, 127.05, 126.47, 123.70, 120.74, 114.22; MS(APCI): 419.80 for C_26_H_17_N_3_OS [M + H^+^].

### 3.3. OLED Fabrication and Characterization

The device substrate was made by using an ITO (indium tin oxide) with the thickness of 150 nm. Then substrate was subjected to ultra-sonication with isopropyl alcohol and deionized water, followed by ultraviolet and ozone treatment. Further device fabrication continued as follow: 4,4′-bis(*N*-phenyl-1-naphthylamino)biphenyl (NPB) was used as hole transporting material (HTM), 1,3-bis(*N*-carbazolyl)benzene (mCP) was used for exciton blocking layer, bis[2-(diphenylphosphino)phenyl] ether oxide (DPEPO) and bis[2-(2-pyridinyl)phenolato]beryllium(II) (Bepp2) host materials doped with 6% fluorescence emitters, 2,2′,2″-(1,3,5-benzinetriyl)-tris(1-phenyl-1-*H*-benzimidazole) (TPBI) was electron transporting material, 8-quinolinolato lithium (Liq) used for hole injecting material and aluminium was used for cathode with thickness of 100 nm. All organic layers were deposited by thermal evaporating system under 5 × 10^−7^ torr pressure (Sunicel plus, Seoul, Republic of Korea). The active area of the devices were 2 mm × 2 mm.

## 4. Conclusions

In summary, we have designed and synthesized four bipolar fluorescence emitters for OLEDs. All four materials were synthesized by using Buchwald-Hartwig amination with excellent yield over 75%. Here, we used oxadiazole acceptor with acridine derivatives (DPAc-OXD, 2DPAc-OXD) and phenothiazine derivatives (PTZ-OXD, 2PTZ-OXD) to construct bipolar fluorescence materials. All four materials exhibited higher thermal stabilities which were expressed by thermal decomposition temperature at 5% weight reduction. In order to investigate the device efficiencies, we have fabricated two different devices with two different host materials Bepp2 (Device 1) and DPEPO (Device 2). The Device 1 performances were considerably higher than that of Device 2. The phenothiazine donor based 2PTZ-OXD emitter revealed the best current and power efficiencies of 10.10 cd/A and 6.88 Lm/W, respectively. The external quantum efficiency of 2PTZ-OXD was 3.99%, while PTZ-OXD showed 2.26%. When we compared the overall performances, 2PTZ-OXD exhibited excellent properties while acridine derivatives based 2DPAc-OXD and DPAc-OXD showed lower efficiencies due to its strong donating power. We found that balance charge donating and withdrawing nature were very important for bipolar fluorescence emitters in OLEDs to enhance the efficiencies.

## Figures and Tables

**Table 1:** Thermal and physical properties of fluorescent emitters.

Dopant	*T_d_* ^a^ (°C)	UV-vis ^b^ (nm)	PL max ^c^ (nm)	HOMO ^d^ (eV)	LUMO ^e^ (eV)	*E_g_* ^f^ (eV)	*E_T_* ^g^ (eV)
PTZ-OXD 2PTZ-OXD DPAc-OXD 2DPAc-OXD	358 419 375 455	376 384 396 402	500 512 435 442	−5.48 −5.48 −5.69 −5.68	−2.18 −2.25 −2.56 −2.60	3.30 3.23 3.13 3.08	2.48 2.42 2.85 2.81

^a^ Decomposition temperature at 5% weight reduction; ^b^ On set UV absorption; ^c^ Photoluminescence emission (PL); ^d^ Highest occupied molecular orbital energy (HOMO); ^e^ Lowest unoccupied molecular orbital energy (LUMO); ^f^ Band gap energy; ^g^ Triplet energy.

**Table 2:** Calculation data of four dopant materials.

Calculation Values	PTZ-OXD	2PTZ-OXD	DPAc-OXD	2DPAc-OXD
S_1_ (eV) ^a^ T_1_ (eV) ^b^ Δ*E*_ST_ (eV) ^c^ D-A rotation(^°^) HOMO (eV) LUMO (eV)	3.71 3.36 0.35 77.05 −5.975 −2.185	3.63 3.34 0.29 78.91 −5.485 −2.255	3.83 3.37 0.46 81.26 −5.695 −2.565	3.78 3.34 0.44 81.70 −5.685 −2.605

^a^ Singlet energy; ^b^ Triplet energy; ^c^ Triplet and singlet energy difference.

**Table 3:** Device characteristics of fluorescent emitters PTZ-OXD, 2PTZ-OXD, DPAc-OXD and 2DPAc-OXD.

Device properties	PTZ-OXD	2PTZ-OXD	DPAc-OXD	2DPAc-OXD
Turn on voltage (V)	4.4 ^a^ 6.0 ^b^	4.2 ^a^ 5.6 ^b^	4.1 ^a^ 6.0 ^b^	4.1 ^a^ 6.2 ^b^
Driving voltage (V)	6.8 ^a^	5.9 ^a^	7.6 ^a^	7.5 ^a^
Current (mA)	0.05 ^a^ 0.05 ^b^	0.03 ^a^ 0.03 ^b^	0.04 ^a^ 0.06 ^b^	0.05 ^a^ 0.10 ^b^
Current efficiency (cd/A)	5.03 ^a^ 4.97 ^b^	9.20 ^a^ 10.10 ^b^	1.30 ^a^ 0.72 ^b^	1.28 ^a^ 0.66 ^b^
Power efficiency (Lm/W)	3.59 ^a^ 2.60 ^b^	6.88 ^a^ 5.67 ^b^	0.99 ^a^ 0.38 ^b^	0.98 ^a^ 0.33 ^b^
EQE (%)	1.94 ^a^	3.38 ^a^	1.84 ^a^	1.81 ^a^
	2.26 ^b^	3.99 ^b^	1.08 ^b^	0.88 ^b^
Luminance (at 1000 cd/m^2^)	1008	1020	1029	992
CIE (x,y)	(0.38, 0.50) ^a^ (0.43, 0.14) ^b^	(0.40, 0.53) ^a^ (0.31, 0.49) ^b^	(0.16, 0.12) ^a^ (0.18, 0.12) ^b^	(0.17, 0.14) ^a^ (0.18, 0.15) ^b^

^a^ Device 1; ^b^ Device 2.
